# Evaluation of Hemp Seed Oils Stability under Accelerated Storage Test

**DOI:** 10.3390/antiox11030490

**Published:** 2022-02-28

**Authors:** Matilde Tura, Diana Ansorena, Iciar Astiasarán, Mara Mandrioli, Tullia Gallina Toschi

**Affiliations:** 1 Department of Agricultural and Food Sciences, Alma Mater Studiorum—Università di Bologna, Viale G. Fanin 40, 40127 Bologna, Italy; matilde.tura2@unibo.it (M.T.); mara.mandrioli@unibo.it (M.M.); tullia.gallinatoschi@unibo.it (T.G.T.); 2 Department of Nutrition, Food Science and Physiology, School of Pharmacy and Nutrition, Universidad de Navarra, Irunlarrea 1, 31008 Pamplona, Spain; iastiasa@unav.es

**Keywords:** hemp seed oil, accelerated storage, volatile compounds, solid-phase microextraction, fatty acids

## Abstract

The interest in hemp seed oil has recently increased, due to the latest regulations which allow its use as food. Hemp seed oil is characterized by a high content of polyunsaturated fatty acids, which are highly prone to oxidation. Accelerated thermal oxidation (60 °C, 18 days) has been applied to nine types of cold-pressed hemp seed oils to monitor the evolution of the samples during oxidative deterioration. The results showed that the only determinations of primary (peroxide value) and secondary (TBARs) oxidation products did not allow a sufficient or correct evaluation of the oxidative changes of hemp seed oils during storage. In fact, samples at the end of the test were primarily characterized by a high presence of oxidation volatile compounds and a significant decrease of antioxidants. Several volatiles identified before the accelerated storage, such as the predominant α-pinene and β-pinene, gradually decreased during the accelerated storage period. On the other hand, aldehydes (hexanal, (*E*)-2-hexenal, heptanal, (*E,E*)-2,4-hexadienal, (*E*)-2-heptenal, (*E,E*)-2,4-heptadienal, (*E,Z*)-2,4-heptadienal, 2-octenal, nonanal, nonenal, 2,4-nonadienal, (*E,E*)- 2,4-decadienal and 2,4-decadienal), ketones (1-octen-3-one, 3-octen-2-one, (*E,E*)-3,5-octadien-2- one and 3,5-octadien-2-one), acids (propionic acid, pentanoic acid, hexanoic acid and heptanoic acid) and 2-pentyl-furan increased during the accelerated storage, as principal markers of oxidation.

## 1. Introduction

*Cannabis sativa* L. is an annual plant that produces small seeds, botanically named “achenes”. Cold pressing of these seeds allows obtaining hemp seed oil, which is mainly used as food [1]. The uses of hemp have been relaunched in the recent years, thanks to the possibility of cultivation of several industrial varieties as well as the wide range of applications and the low impact of its cultivation on the environment [2,3]. In particular, there is great commercial value for seeds and the secondary metabolites of hemp used in the pharmaceutical and cosmetic industries [3]. Data reported by FAO (Food and Agriculture Organization of the United Nations) in 2019 showed that global production of hemp seed has increased in the last 60 years, with a hemp seed production of more than 100,000 tons, while the harvested area decreased, thus indicating the improvement of agronomic practices [3]. This is due to the rapid increase of the hemp seed market every year, as is the demand for hemp products [4]. Hemp seed oil (HSO) is a source of polyunsaturated fatty acids (PUFAs), in particular linoleic acid (18:2 n-6, generally present at 55%) and α-linolenic acid (18:3 n-3, generally present at 20%). Moreover, γ-linolenic acid (18:3 n-6; approximately 1–4%) and stearidonic acid (18:4 n-3; ranges from 0.5–2%) were also identified in this oil [5]. The *Codex Alimentarius* clearly defines cold-pressed oils as “obtained, without altering the oil, by mechanical procedures only, e.g., expelling or pressing, without the application of heat. They may have been purified by washing with water, settling, filtering and centrifuging only” [6]. The cold-pressing process also extracts minor compounds naturally present in hemp, i.e., antioxidants such as phenols and tocopherols [7,8,9]. The evaluation of the total phenolic and tocopherols contents could be useful to evaluate the differences among samples and during storage in terms of antioxidants [10]. On the other hand, it also determines the presence of high chlorophyll content, which is a photosensitive pigment that could affect the quality of hemp seed oil during storage [11,12]. In fact, the presence of chlorophyll as well as the great content of PUFAs, which are highly prone to oxidation, could lead to oxidative degradation during storage of hemp seed oil, also at room temperature due to the low activation energy required [12,13].

In addition, several cannabinoids had been found in hemp seed oil even if the hemp seeds did not contain cannabinoids. In fact, their presence is due to the contact of hemp seeds with the resin located on flowers, leaves or bracts, so they are considered as “impurities” or “contaminants” of hemp seed oil [1]. Although these compounds are present only in small quantities, they have medical interest due to their bioactive activities, such as anti-convulsive and anti-epileptic effects [14]. Cannabinoid acids such as cannabidiolic acid (CBDA) convert to corresponding neutral forms (CBD-cannabidiol) through a decarboxylation reaction that is catalyzed by heat. Hence, the changes of the CBDA/CBD ratio in HSO can be considered as a useful indicator for monitoring HSO storage life [15].

The lipid oxidation process involves a complex series of chemical reactions, which lead to the formation of primary (commonly measured as peroxide value and UV absorbance at 232 nm) and secondary (commonly measured with several indices, such as conjugated diene and triene, *p*-anisidine value and thiobarbituric acid value) oxidation products [12,16,17]. Furthermore, the oxidation process could also be sensory detected; in fact, it determines the formation of off-flavors in the oil, such as rancid [18]. The aroma of the oil is formed by the presence of volatile compounds, which have different odor thresholds, meaning that a high or low concentration of volatile compounds does not directly impact the oil’s sensory quality. The oil flavor could be affected by volatile compounds from the plant and volatiles deriving from chemical changes during storage, such as oxidation. Several classes of volatile compounds could impact the quality deterioration, e.g., aldehydes, ketones, esters and furan derivatives, and specific compounds are usually identified as markers of lipid oxidation, e.g., hexanal and nonanal [18]. It is widely known that the oxidation borne by linoleic acid conducts to the production of hexanal, 2-heptenal, 2-octenal, (*E,Z*)-2,4-decadienal and (*E,E*)-2,4-decadienal, while the oxidation of linolenic acid can give rise to the production of a more significant proportion of (*E,Z*)-2,4-heptadienal and (*E,E*)-2,4-heptadienal [19]. Several volatile compounds that could be formed during lipid oxidation, such as 4-hydroxy-2-hexenal and 4-hydroxy-2-nonenal, which come from the oxidation of ω-3 and ω-6 polyunsaturated groups, also present a harmful impact on human health [20]. One of the primary analytical techniques used for the volatiles analysis is solid-phase microextraction (SPME) because it is rapid, solvent-free and sensitive [17,18,21]. Moreover, oxidation determines the quality degradation of the oil during storage, also from the nutritional point of view [22].

This study aimed to evaluate the changes in the characteristics of hemp seed oils during accelerated thermal oxidation storage, focusing on the modifications in the volatile profile. Moreover, another goal was to determine the main volatile markers of lipid oxidation and freshness in hemp seed oil. The progress of the oxidation was monitored by performing several analyses related to the oxidation and the composition of hemp seed oils. In fact, to the authors’ knowledge, although the interest in hemp seed oil is increasing, there are still few studies in the literature investigating changes during storage, focusing on the volatile profile.

## 2. Materials and Methods

### 2.1. Samples and Accelerated Storage

Nine types of different cold-pressed hemp seed oils (food grade) from the Italian market were employed. Table 1 shows the main information reported on the labels of samples. The seeds’ origin was reported on the labels only as “UE agriculture” or “Extra UE agriculture”, where UE stands for European Union.

Oils were distributed in glass vials (2 g in each vial) and oxidized at 60 °C, as also applied by Gaca et al. (2021) [18], in the oven for a total period of 18 days. In these conditions (*Schaal oven* conditions), one day at 60 °C is equivalent to one month at room temperature. Sampling and analyses were performed every 3 days. The samples were named with a number (from 1 to 9, for the types of oils), and the oxidation time was indicated by “T” followed by the number of days of accelerated oxidation, from T0 (i.e., time 0 or 0 days of accelerated oxidation test) to T18 (i.e., time 18 or 18 days of accelerated oxidation test).

### 2.2. Peroxide Value

Peroxide value (PV) was determined at 510 nm by following the methods reported by Shantha and Decker (1994) [23]. Briefly, an aliquot of sample (corresponding to approximately 10 mg of oil) was transferred to a tube. The residue was dissolved in 5 mL of a mixture of butanol/methanol (2:1). An amount of 25 μL of SCNNH_4_ (7.5 g in 25 mL of distilled water) was added and tubes were vortexed for 4 s. Then, 25 μL of a solution of FeCl_2_ (36 mM in HCl) was added and tubes were vortexed again for 4 s. After 15 min in the dark, absorbance was measured at 510 nm (FLUOStar Omega spectrofluorometric analyzer, BMG Labtechnologies, Offenburg, Germany).

A calibration curve with cumene hydroperoxide was used for quantification (*y* = 7.9473*x* + 0.0363; *r*^2^ = 0.9983). Results were expressed as mEqO_2_/kg of oil.

### 2.3. Determination of Thiobarbituric Acid Reactive Substances (TBARs)

TBARs value was determined at 532 nm according to Maqsood and Benjakul (2010), with slight modifications [24]. Briefly, an aliquot of 0.25 g of oil was added with 0.4 mL of distilled water, 1 mL of thiobarbituric acid and 10 μL of butylhydroxytoluene, and then it was vortex for 30 s. After 15 min in a hot bath (100 °C) and 10 min in a cold-water bath, 2 mL of cyclohexanone and 0.5 mL of ammonium sulphate were added. Then, the samples were centrifuged for 10 min at 4000 rpm and an absorbance of 200 μL was read at 532 nm (FLUOStar Omega spectrofluorometric analyzer, BMG Labtechnologies, Offenburg, Germany).

A calibration curve with 1,1,3,3-tetrahydroxypropane was used for the quantification (*y* = 30850*x* + 0.1866; *r*^2^ = 0.9904). Results were expressed in mg of malonaldehyde (MDA)/kg of sample. Moreover, data were also recorded at 390 nm, in order to evaluate a possible interference of aldehydes, as reported by Poyato et al. (2014) [19].

### 2.4. Determination of Total Phenols

Total phenolic content was determined at 765 nm, according to the method reported by Singleton and Rossi (1965) [25]. One gram of oil was added with 20 mL of *n*-hexane and 20 mL of methanol/water (80:20). After the separation of the two phases, the lower phase was recovered and dried with a rotavapor at 40 °C. Then, the dried phase was recovered with 5 mL of distilled water. An amount of 100 μL of the samples was added to 7.9 mL of distilled water, with 500 μL of Folin–Ciocalteu reactive. After 2 min of waiting, 1.5 mL of Na_2_CO_3_ saturated solution was added and then the samples were stored for 2 h in the dark. After 2 h, the absorbance of 300 μL was read at 765 nm (FLUOStar Omega spectrofluorometric analyzer, BMG Labtechnologies, Offenburg, Germany). A calibration curve with gallic acid was used for the quantification (*y* = 0.9423*x* + 0.0077; *r*^2^ = 0.9998). Results were expressed as mg of gallic acid/kg of oil.

### 2.5. Fatty Acid Composition

The determination of fatty acid composition was conducted according to the AOAC official method (2002) [26]. A PerkinElmer Clarus 500 gas chromatograph (Perkin Elmer, Shelton, USA) equipped with a capillary column SPTM-2560 (100 m × 0.25 mm × 0.2 μm, Supelco, Bellefonte, USA) and flame ionization detector was used. The GC-FID conditions were set as reported by Gutiérrez-Luna et al. (2020) [27]. The identification of fatty acid methyl ester (FAME) was made by comparing the retention times of the peaks in each sample with those of standard pure compounds. Individual methylated standards from Sigma-Aldrich (St. Louis, MO, USA) were used.

### 2.6. Volatile Profile

Volatile compounds were analyzed by headspace solid-phase microextraction (HS-SPME) combined with gas chromatography–mass spectrometry (GC–MS) by following the method reported by Gayoso et al. (2017) [28], with some modifications. The SPME fiber coating used was Divinylbenzene/Carboxen/Polydimethylsiloxane (DVB/CAR/PDMS) (50/30 µm film thickness, Supelco, Bellefonte, USA). Then, 2 g of oil was weighed into a 25 mL headspace vial and capped with a rubber cap. The sample was equilibrated at 40 °C for 15 min and the adsorption time, with the fiber exposed to the headspace of the sample, was 60 min at the same temperature. During SPME sampling, the vials were not stirred. The desorption time for the fiber in the injection port of the gas chromatograph was 30 min. The GC–MS instrumentation used was GC 6890 N coupled to a mass selective 5973 detector (Agilent Technologies, Santa Clara, CA, USA). Volatiles were separated using a capillary column HP-5MS, 5% phenyl methyl siloxane (30 m long × 0.25 mm inner diameter × 0.25 μm film thickness, Agilent Technologies, Santa Clara, CA, USA). Chromatographic conditions were the same used by Gutiérrez-luna et al. (2022) [29]: the oven temperature was held for 5 min at 42 °C, then increased to 120 °C at 3 °C min-1 and to 250 °C at 10 °C min^−1^ (5 min hold); injector temperature, 270 °C; detector temperature 280 °C; ion source temperature, 230 °C; quadrupole mass analyzer temperature, 150 °C. Helium was used as carrier gas at 1 mL min^−1^. The mass spectrometer was operated by electronic impact at 70 eV, and ions were scanned over the *m/z* range of 33–350 at a rate of 4.43 scan/s. Prior to every SPME extraction, the cleanness of the fiber was controlled by performing a blank and checking the absence of peaks in the chromatogram.

The identification of each peak was made taking into account the Kovats index (KI) reported in the literature [30] and comparing their mass spectra with the one of a commercial library (Wiley 275.L, Mass Spectral Database).

KI was calculated for each detected peak using the following formula:

$$KI=\left[100*\left(tR\left(i\right)-\frac{tR\left(z\right)}{tR\left(z+1\right)}-tR\left(z\right)\right)\right]+100z$$

where:

*z* is the number of carbon atoms in alkane *z*;

*tR*(*i*) is the retention time of compound *i*;

*tR*(*z*) is the retention time of alkane *z*;

*tR*(*z* + 1) is the retention time of alkane *z* + 1.

A semi-quantitative analysis was performed by measuring the area of the peaks, integrating the total ion current of the spectra. In the case of overlapping peaks, the quantification of the corresponding compound was carried out by a specific ion and considering the relative proportion in which this ion is present in each compound. Results were expressed in area counts, as area/sample weight (g) × 10^3^.

### 2.7. Tocopherols

The determination of tocopherols was performed by HPLC (high performance liquid chromatography), applying the method reported in ISO/FDIS 9936:2016 [31], UNI/TS 11825:2021 [32] and by Tura et al. (2019) [33]. Briefly, 0.5 g of oil were weighted in 10 mL flask and brought to volume with isopropanol. Identification of tocopherols was performed by injecting standard of γ-tocopherol (CAS number 54-28-4; Sigma-Aldrich, MI, USA) and α-tocopherol (CAS number 119-13-1; Sigma-Aldrich, MI, USA). Quantification was carried out using a calibration curve constructed with the external standard method, injecting solutions of known concentration in the range of 0.5–100 mg/mL for both α-tocopherol (*y* = 38.811*x* + 41.366; *r*^2^ = 0.998) and γ-tocopherol (*y* = 123.04*x* + 109.1; *r*^2^ = 0.9985).

### 2.8. Cannabinoids

A total of 500 mg of oil was weighed in a 10 mL flask, solubilized, brought to volume with isopropanol, vortexed for 1 min and placed in an ultrasonic bath (Branson 2150, Marshall Scientific, Hampton, NH, USA) for 10 min. Next, the solution was filtered through a 0.45 μm nylon filter. The determination of cannabinoids was performed by using an HPLC-UV (HPLC Cannabis Analyzer for Potency Prominence-i LC-2030C equipped with a reverse phase C18 column, Nex-Leaf CBX Potency 150 4.6 mm, 2.7 m with a guard column Nex-Leaf CBX 5 4.6 mm, 2.7, UV detector and acquisition software LabSolutions version 5.84; Shimazu, Kyoto, Japan), following the method proposed by Mandrioli et al. (2019) [34]. Quantification was carried out using a calibration curve constructed with an external standard, injecting solutions (Phytocannabinoid Mixture 10 (CRM), Cayman Chemical, Ann Arbor, Michigan, USA) of known concentration in the range 0.05–5 μg/mL (CBDA *y* = 15844*x* − 2893.9, *r*^2^ = 0.9997; CBGA *y* = 16409*x* − 2591.7, *r*^2^ = 0.9996; CBG *y* = 14113*x* − 2002.3, *r*^2^ = 0.9994; CBD *y* = 12877*x* − 281.01, *r*^2^ = 0.995).

### 2.9. Data Analysis

Samples were analyzed in triplicate, and the results are shown as mean ± standard deviation. Only for the fatty acids profile, samples were prepared in duplicate and each replicate was injected twice. Data were statistically analyzed by using the software XLSTAT Addinsoft (2018.XLSTAT statistical and data analysis solutions, version 2018.1.1. Addinsoft, Paris, France. https://www.XLSTAT.com, (accessed on 20 January 2022).

Results of the peroxide value, TBARs, total phenolic compound, tocopherols and cannabinoids were subjected to one-way ANOVA (Tukey’s HSD, *p* < 0.05) in order to highlight differences in the samples during the accelerated storage. Moreover, a two-way ANOVA with interactions (peroxide valuex storage time) (*p* < 0.0001) was performed on the peroxide data. Volatile compounds were graphically represented as heat map. Furthermore, in order to select specific volatile compounds as markers of oxidation or freshness, a Pearson correlation matrix among volatiles detected in all the 9 samples during the accelerated test and the storage times was carried out.

Finally, principal component analysis (PCA) was performed including volatiles detected in all the nine samples at each point of analysis (from time zero to time eighteen).

## 3. Results

### 3.1. Peroxide Value

**Figure 1:**
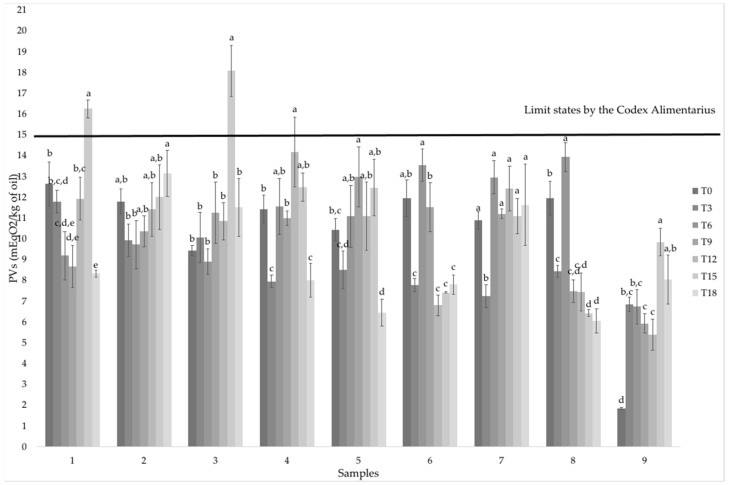
Results of the peroxide value expressed as mEqO2/kg of oil during the accelerated oxidation test (from T0 to T18, during 18 days of heating at 60 °C). Data are reported as mean ± standard deviation of 3 independent replicates. Different letters indicate statistically significant differences among Peroxide Values at different oxidation times for each sample (one-way ANOVA, Tukey’s HSD, *p* < 0.05).

All the samples, at time zero, presented PVs lower than the maximum value reported in the *Codex Alimentarius* for cold-pressed vegetable oils, equal to 15 mEqO2/kg of oil. Samples 1 and 3, after 15 days of accelerated oxidation, showed PVs higher than the limit set by the Codex Stan 210-1999 [6] (Figure 1).

Moreover, interaction between the content of peroxide in the samples and oxidation time was evaluated and it showed a statistical significance (two-way ANOVA with interactions, *p* < 0.001), highlighting the influence of the storage time on this parameter related to the oxidative state.

### 3.2. TBARs

The level of lipid oxidation in the hemp seed oils was also evaluated by measuring the thiobarbituric acid-reactive substances (TBARs), before the accelerated oxidation test (time 0/T0) and after 18 days of oxidation in the oven (time 18/T18). TBARs generally reflect the level of the secondary products from lipid peroxidation, with a positive association with lipid peroxidation [35]. At the end of the accelerated oxidation period (after 18 days), the TBARs values decreased in all but one (sample six) types of hemp seed oil (Table 2).

### 3.3. Total Phenolic Compounds

As reported in Table 3, the total phenolic content decreased during the accelerated oxidation. In fact, at the end of the accelerated storage period (i.e., 18 days at 60 °C), all the samples showed a lower phenolic content in comparison to time zero (i.e., before the heating in the oven), although samples four and eight did not show statistically significant differences (one-way ANOVA, *p* < 0.05, Tukey’s HSD).

### 3.4. Fatty Acids

The main fatty acids were: linoleic acid (ranged from 46.24 g/100 g to 51.25 g/100 g at time zero and from 46.25 g/100 g to 53.13 g/100 g at time 18), followed by α-linolenic acid (from 10.61 g/100 g to 17.03 g/100 g at time zero and from 9.75 g/100 g to 16.82 g/100 g at time 18) and oleic acid (from 7.05 g/100 g to 13.10 g/100 g at time zero and from 8.09 g/100 g to 13.07 g/100 g at time 18). Those results are in line with what is reported in the literature [4,36,37]. Moreover, as described in previous studies [5,38], the presence of γ-linolenic acid and stearidonic acid was also detected in all the samples (Table S1).

### 3.5. Volatile Profile

**Figure 2:**
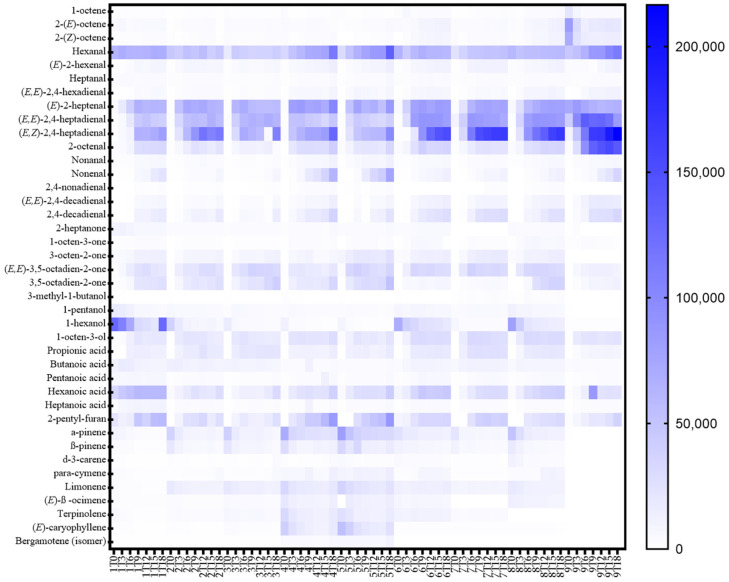
Heat map comparing the content of the volatile compounds in the nine types of hemp seed oils during the accelerated storage test.

The volatile compounds detected during the accelerated storage in the nine types of oils are reported in Figure 2 and in Table S2 (detailed quantification). Several compounds related to oxidation, such as 2,4-nonadienal, (*E,E*)-2,4-decadienal and (*E,Z*)-2,4-decadienal, were observed in the samples after 3, 6, 9, 12, 15 and 18 days of accelerated storage, while they were not always detected at the beginning (time zero).

In Table 4, the correlation matrix (Pearson) among the accelerated storage time and volatiles and the relative *p*-values is reported.

### 3.6. Tocopherols

The tocopherols detected in all the samples were α-tocopherol and γ-tocopherol. The results are shown in Table 5 and are in accordance with the literature [39,40,41], except for sample nine. In fact, sample nine showed a significantly higher content of α-tocopherol with respect to the other samples. According to the literature, the main tocopherol naturally present in hemp seed oils is γ-tocopherol, while the main one detected in sample nine was α-tocopherol.

### 3.7. Cannabinoids

**Figure 3:**
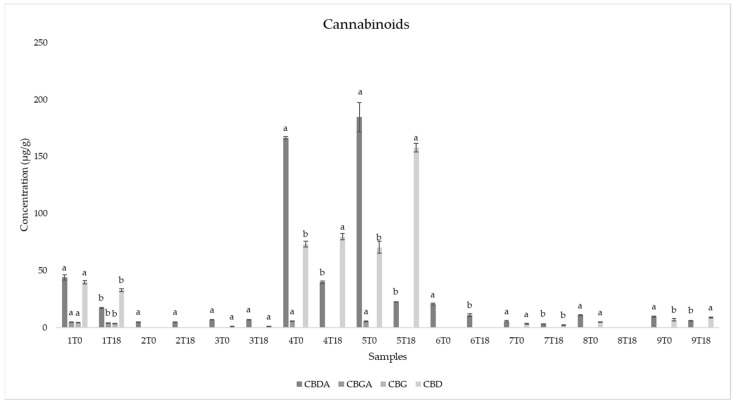
Cannabinoid content (µg/g) in the nine hemp seed oils at time 0 (T0) and time 18 (T18) of the accelerated oxidation test. Different letters between time 0 (T0) and time 18 (T18) in the same samples and for the same cannabinoid indicate statistically significant differences (one-way ANOVA, Tukey’s HSD, *p* < 0.05).

The main cannabinoid present in all the samples was cannabidiolic acid (CBDA). Several samples also showed the presence of cannabidiol (CBD) and cannabigerolic acid (CBG), while only in sample one was cannabigerol (CBG) found. As reported in Figure 3, the content of CBDA decreased from time zero to time 18, in all the samples. On the contrary, the concentration of CBD increased in several samples, such as in samples four and five, for example. Those two samples also showed the highest content of cannabinoids with a concentration of CBDA equal to 166.46 ± 1.01 μg/g in sample four at T0, 184.62 ± 12.85 μg/g in sample five at T0. Sample five also showed a great increase in terms of CBD from time zero to time 18 (passing from 70.43 ± 5.31 μg/g at time zero to 157.95 ± 3.70 μg/g at time 18).

## 4. Discussion

### 4.1. Oxidative State and Composition of the Hemp Seed Oils at the Initial Stage of the Accelerated Oxidation Test

Before starting the accelerated oxidation test, the initial oxidative status and composition of the samples were evaluated (Table S3). The results obtained for PVs at time zero are in accordance with several authors in the literature for hemp seed oil [12,40,42,43]. Most of the samples showed similar values (9.43–12.62), with only sample type nine showing very low values (1.83). Sample nine also showed the highest TBARs values and the highest presence of aldehydes, which, representing secondary oxidation products [19], could highlight a bad oxidative state of the oil that could also be in relation to the low PV value. In fact, it is well known that peroxides are not stable compounds, and generally, their concentration in the oils increases until it reaches a maximum value and then decreases as these degrade to secondary oxidation products [44]. The highest content of ketones, acids and furans was detected in sample one. Since also those compounds are generally related to the oxidation [45,46], their presence could indicate a worse oxidation state of this oil compared to the others. In addition, the lowest content of terpenes was detected in the head space of sample nine. According to the literature, most of the volatile compounds linked to oxidation have a low odor threshold, and the main contribution to rancid defects comes from aldehydes [45]. However, terpenes can positively contribute to the aroma of hemp seed oil, bringing aromas such as hop, pine, lime and spice [47]. For these reasons, sample nine could be mainly characterized by an off flavor related to oxidation.

The main fatty acids (oleic, linoleic, α- linolenic and γ-linolenic acids) were also evaluated in order to highlight differences among samples. In particular, the principal fatty acid was C18:2 n-6 (linoleic acid), which showed the lowest value in sample nine.

Phenolics greatly affect the stability and nutritional characteristics of oil samples and might prevent their deterioration through quenching of radical reactions responsible for lipid oxidation [48]. The total phenolic content was in accordance with what was found in the literature [35,49,50]. The highest phenolic content was detected in sample nine, which was significantly different from all the other oils (Table 3).

A high content of γ-tocopherol characterizes hemp seed oil, around 80–90% of the total amount of tocopherols (80–150 mg/100 g) in comparison with many edible oils (e.g., olive oil), which is a naturally present antioxidant in this oil [41]. The content of γ-tocopherol was in accordance with the literature for all the samples [51], while the content of α-tocopherol detected in sample nine was greatly higher than previous studies [50,51,52].

**Figure 4:**
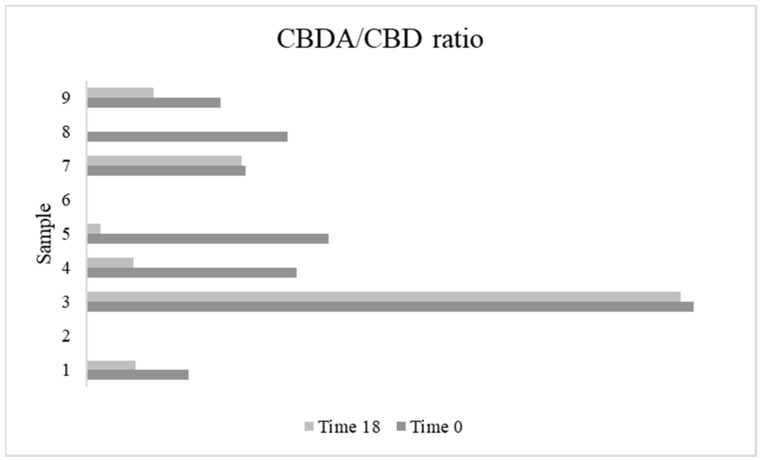
Ratio between cannabidiolic acid (CBDA) and cannabidiol (CBD) at the beginning (time 0/T0) and at the end (time 18/T18) of the accelerated oxidation test.

Finally, the ratio between cannabidiolic acid (CBDA) and cannabidiol (CBD) (Figure 4) was also considered, since it could be a helpful index for hemp seed oil storage. This is related to the decarboxylation reaction, which determines the conversion of CBDA (i.e., the acid form naturally produced by the hemp plant) to CBD (i.e., the neutral form) [15]. Only samples two and six showed the presence of neither CBDA nor CBD, and in sample three, the higher ratio of CBDA/CBD was detected. However, the presence of cannabinoids is strictly related to cross-contamination with flowers/leaves or a bad selection of the bracts [1]. For this reason, even if the ratio of CBDA/CBD could be a helpful index, it cannot be considered alone: some hemp seed oils do not show the presence of cannabinoids (Figure 3 and Figure 4). Several parameters evaluated and discussed in previous lines highlighted that the initial state and composition of sample nine were very different from the other oils tested.

### 4.2. Evolution of the Oxidative State and Composition of the Hemp Seed Oils during the Accelerated Heating Test

As reported in Figure 1, the PVs increased during the accelerated oxidation test, and then they decreased at the end. Only samples two, seven and nine showed an increase in the PVs until the end accelerated storage. Regarding TBARs, data showed a lower value at time 18 in comparison to time zero in all samples, except in sample six, where the decrease was not statistically significant (Table 2). Poyato et al. (2014) [19] reported that the presence of a high content of aldehydes during storage gives rise to the formation of yellow chromophores, determining a significant absorbance at 390 nm instead of at 532 nm, which is the traditional wavelength used for measuring TBARs. This could explain the decrease of TBARs registered; in fact, the absorbance at 390 nm was also higher than at 532 nm in our case (Figure S1).

The total phenolic content decreased from time zero to the end of the storage period in seven out of the nine types of samples (Table 3), with sample nine maintaining the highest phenolic content at the end of the treatment. These decreases (mean value 86%) could be related to the oxidation process revealed by the analysis of volatile compounds and the destruction of the phenols acting as antioxidants. Regarding these volatiles, aldehydes significantly increased during the heating treatment in all samples (*p* < 0.05) (Table 6). In fact, increments between four and 13-fold were noticed for this volatile class linked to lipid oxidative processes.

Among aldehydes, two saturated species were detected, heptanal and nonanal (Figure 2 and Table S2), showing an increment during heating, especially in the case of nonanal. Their presence could be related to the decomposition of hydroperoxides formed by the autoxidation of oleic acid [21]. (*E*)-2-hexenal was also present at the beginning of the storage in all the samples and increased during the accelerated oxidation test; thus, it could be related to two different phenomena: the linoleate autoxidation decomposition and the enzymatic oxidation of linolenic acid [21,53,54]. Hemp seed oils are rich in ω-3 and ω-6 fatty acids (Supplementary Material Table S1) [50,55] and the secondary products of lipid oxidation, such as volatiles, greatly depend on the fatty acid substrate [56]. According to Nogueira et al. (2019) [56], several volatile compounds related to the oxidation of ω-3 rich oils are (*E,E*)-2,4-heptadienal, (*E,E*)-3,5-octadien-2-one and (*E,E*)-2,4- decadienal, which are potentially toxic volatiles, while of the ω-6 rich oils are (*E,E*)-2,4-heptadienal and nonanal; in fact, those compounds were detected in the nine types of oxidized oils (Table S2 and Figure 2).

On the other hand, terpenes, typically present in fresh oils, decreased between 1.3 and 2.5-fold during storage (Table 6). The most abundant ones were α-pinene and β-pinene, and this result is in line with Zhou et al. (2017) [57]. Moreover, as reported in Table S2, sample type nine showed a very different terpenes profile compared to the other samples. In fact, α-pinene was the only terpene detected in this type of oil, while for the others β-pinene, δ-3-carene, ρ-cymene, limonene, (*E*)-β -ocimene, terpinolene and (*E*)-caryophyllene were also identified.

Regarding tocopherols, the content of α-tocopherol during oils storage usually decreases, in particular if the overall oxidative status of the oil is not good (e.g., high PVs), resulting in a decrease of the nutritional value of the oil [58]. In all the samples, α-tocopherols were detected only at time zero (before starting the accelerated oxidation test), while γ-tocopherol was always identified, and it showed a significant decrease (Table 5). The main decrease was noticed between 3–9 days, depending on the samples, and then the amounts were stable until the eighteenth day. This reduction of tocopherols in the matrix could be related to the antioxidant activity of those compounds, slowing down the oxidation process in the oil [41].

Results showed that the most abundant cannabinoid was CBDA (Figure 3), which is in line with previous literature [1,15]. This cannabinoid, found in all the samples, showed a reduction during the accelerated storage test. According to Pratap Singh et al. (2020) [15], this decrease occurred due to the decarboxylation reaction of CBDA. Citti et al. (2018) [1] hypothesized that, at a temperature under 100 °C, the decarboxylation of CBDA leads only to the formation of CBD, increasing this cannabinoid and leading to a nearly constant sum of CBDA + CBD, while at a temperature above 100 °C it also determines the formation of unknown products or the evaporation of the neutral cannabinoid. For some samples, our results showed different trends in comparison to Citti et al. (2018) [1]: in particular, a decrease of CBDA with no increase in terms of CBD was registered in samples one, four, six and eight, even if the temperature of the accelerated oxidation was 60 °C. Samples two, three and seven showed a constant sum of CBDA+CBD without substantial changes in their content, while in samples five and nine a decrease of CBDA with a consequent increase of CBD content was highlighted. Moreover, a decrease in the CBDA/CBD ratio was detected in the majority of the samples, and only for sample three were no differences found (Figure 4). Samples two and six did not show the presence of CBD, and for this reason the CBDA/CBD ratio was not reported.

### 4.3. Evaluation of the Oxidation and Freshness Volatiles Markers

In order to select volatile compounds as possible markers of oxidation and freshness during the accelerated storage period, the amount of each compound was monitored during the test and correlated with the storage time.

Table 4 shows the Pearson value, *p*-values and significance of the correlation between the area counts ×10^3^ of each compound and the storage time (reported in days). All the aldehydes detected showed a positive and significant correlation with the accelerated oxidation time, confirming them to be markers of oxidation of edible oils [18,19,21,53,54]. Moreover, several ketones (1-octen-3-one, 3-octen-2-one, (*E,E*)-3,5-octadien-2-one and 3,5-octadien-2-one), acids (propionic acid, pentanoic acid, hexanoic acid and heptanoic acid) and furans (2-pentyl-furan) showed a positive correlation with storage time. Moreover, those compounds were reported to be related to the oxidative phenomena in edible oils [59,60]. Among them, the principal oxidation volatile markers were (*E*)-2-hexenal, (*E,E*)-2,4-hexadienal, (*E,Z*)-2,4-heptadienal, nonanal, (*E,E*)-2,4-decadienal, 2,4-decadienal and 2-pentyl-furan, showing the highest R Pearson values.

On the other hand, two terpenes (α-pinene and β-pinene) were inversely related to the accelerated oxidation time, thus indicating them as possible markers of the freshness of the cold-pressed hemp seed oils. 1-pentanol and 1-hexanol were also inversely correlated with storage time (days), suggesting that the accelerated storage conditions could also determine the further oxidation of the alcohols, according to Vichi et al. (2003) [21].

**Figure 5:**
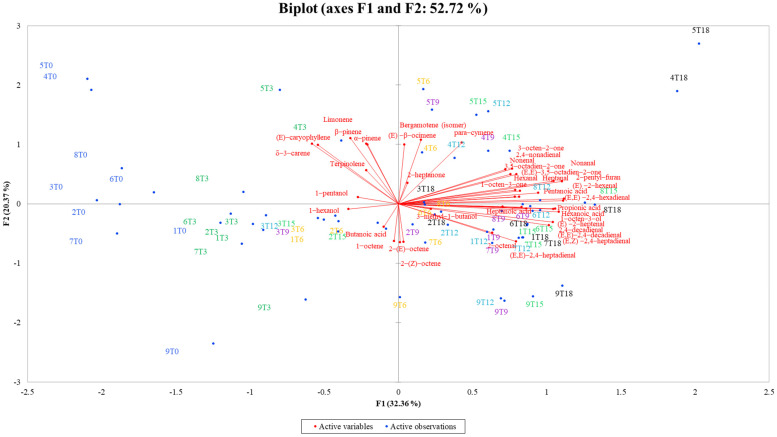
Representation of the principal component analysis (PCA) for volatiles identified in all the samples at each time of analysis (0, 3, 6, 9, 12, 15 and 18 days of accelerated storage). Observations are shown in blue dots and variables are shown in red font.

Figure 5 reports the volatile compounds detected at each time of the storage period for the nine types of oils. At time zero (before starting the accelerated oxidation), samples are separated from all the other storage times, suggesting that they presented a very different volatile profile than the oxidized samples. Moreover, it is possible to notice that the samples at time three (3 days of accelerated oxidation test) are also grouped in the same PCA quadrants as T0 and separated from the others, showing an intermediate oxidation status. In fact, they were mainly characterized by 1-octene, 1-pentanol, 1-hexanol, butanoic acid, α-pinene, β-pinene, limonene, terpinolene and (*E*)-caryophyllene. On the contrary, samples from time six (6 days of accelerated oxidation test) to time 18 (18 days of accelerated oxidation test) were not separated among them and those clustered with aldehydes, several ketones and acids and 2-pentyl-furan (Figure 5).

## 5. Conclusions

During the accelerated storage test of the nine types of commercial hemp seed oils (60 °C for 18 days) selected as representatives of different qualities available on the market, different rates of the oxidative process were highlighted. In particular, one hemp seed oil, even before the start of the accelerated oxidation test, showed a high amount of secondary oxidation products (TBARs, specific volatiles), as it was already oxidized. In general, for all samples, the rise of a number of volatile compounds was found to be highly related with the progress of the oxidative process. It consisted of (*E*)-2-hexenal, (*E*,*E*)-2,4-hexadienal, (*E*,*Z*)-2,4-heptadienal, nonanal, (*E*,*E*)-2,4-decadienal, 2,4-decadienal and 2-pentyl-furan. The increase of these compounds can be considered inversely proportional to the freshness of the hemp oil, as is the decrease of peculiar naturally occurring terpenes (in particular α-pinene and β-pinene). Tocopherols and total phenols, acting as antioxidants, also registered a remarkable decrease during the accelerated storage test (i.e., an average reduction of 86% of antioxidant compounds). By the correlation between peroxide values, TBARs, volatiles, phenols and cannabinoids, it was highlighted that the sole determinations of traditional parameters such as peroxide value and TBARs were not enough to appreciate the oxidative changes occurring during the accelerated storage. This is related with the known instability of the peroxides and to the growing interferers, due to the increase of the aldehydes during the course of oxidation, in the determination of TBARs. On the contrary, on the eighteenth day (end of the test) the samples were mainly characterized by a number of highly representative oxidative volatiles markers, mainly aldehydes, which were very low or even absent in fresh samples.

## Figures and Tables

**Table 1:** Samples used for the experimentation. Information was reported on the label.

Sample	Farming System	Technological Information	Seeds Origin
1	Organic	Cold-pressed	Italy
2	Organic	Cold-pressed	UE-Extra UE
3	Organic	Cold-pressed	Extra UE
4	Organic	Cold-pressed	UE
5	Conventional	Cold-pressed	UE
6	Organic	Cold-pressed	Extra UE
7	Organic	Cold-pressed	UE
8	Organic	Cold-pressed	UE
9	Organic	Cold-pressed	UE

UE: European Union.

**Table 2:** Results of the TBARs expressed as mg MDA/kg of oil at the beginning (T0) and at the end (T18) of the accelerated oxidation test. Data are reported as mean ± standard deviation of 3 independent replicates. Different letters (a, b) in rows indicate statistically significant differences (one-way ANOVA, *p* < 0.05, Tukey’s HSD).

Sample	T0 mg MDA/kg of Oil	T18 mg MDA/kg of Oil
1	11.28 ± 0.69 ^a^	9.62 ± 0.74 ^b^
2	11.04 ± 1.58 ^a^	9.06 ± 0.47 ^b^
3	13.27 ± 1.78 ^a^	8.50 ± 0.66 ^b^
4	11.56 ± 1.70 ^a^	9.65 ± 1.42 ^b^
5	11.54 ± 0.28 ^a^	8.12 ± 1.19 ^b^
6	11.37 ± 1.57 ^a^	10.25 ± 1.14 ^a^
7	12.25 ± 1.15 ^a^	7.17 ± 0.73 ^b^
8	12.01 ± 0.97 ^a^	9.98 ± 1.32 ^b^
9	17.15 ± 0.83 ^a^	6.87 ± 0.99 ^b^

**Table 3:** Results of the determination of total phenols expressed as mg gallic acid/kg of oil. Data are reported as mean ± standard deviation of 3 independent replicates. Different letters (a, b) in rows indicate statistically significant differences (one-way ANOVA, *p* < 0.05, Tukey’s HSD).

Sample	T0 mg Gallic Acid/kg of Oil	T18 mg Gallic Acid/kg of Oil
1	50.37 ± 2.03 ^a^	22.14 ± 1.78 ^b^
2	106.50 ± 8.80 ^a^	16.95 ± 0.82 ^b^
3	45.58 ± 2.71 ^a^	11.28 ± 1.15 ^b^
4	15.39 ± 0.79 ^a^	12.76 ± 1.16 ^a^
5	16.75 ± 0.41 ^a^	10.89 ± 0.28 ^b^
6	30.76 ± 6.60 ^a^	7.90 ± 0.21 ^b^
7	25.70 ± 1.07 ^a^	7.10 ± 0.49 ^b^
8	12.08 ± 1.96 ^a^	11.80 ± 2.25 ^a^
9	186.78 ± 4.57 ^a^	60.10 ± 0.63 ^b^

**Table 4:** Correlation matrix (Pearson) and relative *p*-values (Pearson) among volatiles detected during the accelerated storage of nine types of cold-pressed hemp seed oils analyzed at 7 different times (0, 3, 6, 9, 12, 15, 18 days).

Volatile Compounds	*R*	*p*-Values (Pearson)
1-Octene	−0.094	0.464
2-(*E*)-Octene	−0.066	0.608
2-(*Z*)-Octene	−0.054	0.675
Hexanal	0.482	<0.0001
(*E*)-2-hexenal	0.843	<0.0001
Heptanal	0.600	<0.0001
(*E,E*)-2,4-Hexadienal	0.708	<0.0001
(*E*)-2-Heptenal	0.640	<0.0001
(*E,E*)-2,4-Heptadienal	0.488	<0.0001
(*E,Z*)-2,4-Heptadienal	0.752	<0.0001
2-Octenal	0.398	0.001
Nonanal	0.734	<0.0001
Nonenal	0.615	<0.0001
2,4-Nonadienal	0.582	<0.0001
(*E,E*)-2,4-Decadienal	0.719	<0.0001
2,4-Decadienal	0.809	<0.0001
2-Heptanone	0.004	0.973
1-Octen-3-one	0.326	0.009
3-Octen-2-one	0.516	<0.0001
(*E,E*)-3,5-Octadien-2-one	0.559	<0.0001
3,5-Octadien-2-one	0.667	<0.0001
3-Methyl-1-butanol	0.099	0.439
1-Pentanol	−0.278	0.027
1-Hexanol	−0.253	0.045
1-Octen-3-ol	0.619	<0.0001
Propionic acid	0.648	<0.0001
Butanoic acid	−0.059	0.648
Pentanoic acid	0.547	<0.0001
Hexanoic acid	0.546	<0.0001
Heptanoic acid	0.447	0.000
2-Pentyl-furan	0.741	<0.0001
α-Pinene	−0.417	0.001
β-Pinene	−0.381	0.002
δ-3-Carene	−0.167	0.191
*para*-Cymene	0.254	0.045
Limonene	−0.209	0.101
(*E*)-β-Ocimene	−0.063	0.626
Terpinolene	−0.173	0.175
(*E*)-Caryophyllene	−0.192	0.131
Bergamotene (isomer)	0.043	0.740

**Table 5:** Tocopherols content in the nine types of hemp seed oil samples during the accelerated oxidation test. Results are reported as mean ± standard deviation of three replicates and expressed as µg/g. Different letters (a–e) for each tocopherol in each sample indicate statistically significant differences (one-way ANOVA, Tukey’s HSD, *p* < 0.05) during the accelerated storage.

Samples	Time (Days)													
	0		3		6		9		12		15		18	
	α-tocopherol	γ-tocopherol	α-tocopherol	γ-tocopherol	α-tocopherol	γ-tocopherol	α-tocopherol	γ-tocopherol	α-tocopherol	γ-tocopherol	α-tocopherol	γ-tocopherol	α-tocopherol	γ-tocopherol
1	32.09 ± 0.78 ^a^	641.91 ± 29.25 ^a^	-	350.13 ± 5.74 ^b^	-	83.08 ± 2.39 ^c^	-	76.23 ± 1.41 ^c^	-	69.93 ± 1.53 ^c^	-	69.59 ± 1.68 ^c^	-	70.38 ± 1.80 ^c^
2	28.28 ± 0.85 ^a^	568.68 ± 39.34 ^a^	-	65.86 ± 0.76 ^b^	-	56.25 ± 0.30 ^b^	-	60.95 ± 1.39 ^b^	-	54.83 ± 1.09 ^b^	-	58.34 ± 2.40 ^b^	-	60.03 ± 1.88 ^b^
3	45.98 ± 4.15 ^a^	569.99 ± 16.46 ^a^	-	65.62 ± 0.63 ^b^	-	65.48 ± 0.41 ^b^	-	64.76 ± 0.30 ^b^	-	64.27 ± 1.21 ^b^	-	65.74 ± 2.08 ^b^	-	65.65 ± 2.07 ^b^
4	21.02 ± 0.28 ^a^	376.28 ± 10.72 ^a^	-	40.40 ± 0.84 ^b^	-	44.64 ± 1.68 ^b^	-	46.15 ± 0.73 ^b^	-	47.36 ± 0.73 ^b^	-	49.03 ± 3.46 ^b^	-	50.28 ± 1.14 ^b^
5	41.53 ± 3.09 ^a^	650.52 ± 14.68 ^a^	-	103.65 ± 2.90 ^b^	-	65.23 ± 3.58 ^c^	-	73.80 ± 1.72 ^c^	-	68.63 ± 0.83 ^c^	-	72.57 ± 3.44 ^c^	-	67.71 ± 1.30 ^c^
6	65.92 ± 4.86 ^a^	906.63 ± 65.75 ^a^	-	301.72 ± 3.88 ^b^	-	212.29 ± 8.22 ^c^	-	200.12 ± 3.34 ^c^	-	209.57 ± 5.87 ^c^	-	195.09 ± 6.47 ^c^	-	195.57 ± 8.28 ^c^
7	59.30 ± 1.46 ^a^	622.24 ± 12.12 ^a^	-	79.69 ± 10.22 ^b^	-	66.57 ± 1.61 ^b^	-	73.37 ± 1.31 ^b^	-	70.54 ± 0.96 ^b^	-	85.92 ± 5.93 ^b^	-	86.53 ± 1.16 ^b^
8	49.81 ± 0.63 ^a^	893.81 ± 7.78 ^a^	-	384.12 ± 4.77 ^b^	-	300.00 ± 5.10 ^c^	-	233.05 ± 11.29 ^d^	-	206.03 ± 8.11 ^e^	-	207.02 ± 7.70 ^e^	-	237.66 ± 8.34 ^d^
9	1095.62 ± 45.21^a^	816.31 ± 53.49 ^a^	-	327.68 ± 3.27 ^b^	-	83.29 ± 2.46 ^c^	-	59.30 ± 1.68 ^c^	-	55.50 ± 1.75 ^c^	-	51.84 ± 1.73 ^c^	-	53.52 ± 0.59 ^c^

**Table 6:** Comparison of the volatile classes before submitting samples to the accelerated test (T0) and at the end of the oxidation at 60 °C (T18). Different letters (a–h) indicate statistically significant differences among samples, for each volatile class and storage time. Different uppercase letters (A,B) indicate, for each sample and volatile class, statistically significant differences between T0 and T18 (one-way ANOVA, *p* < 0.05, Tukey HSD).

Volatile Classes (Area Counts × 10^3^)	Alkenes	Aldehydes	Ketones	Alcohols	Acids	Furans Derivatives	Terpenes
1 T0	7898 ^b;B^	79,310 ^b;B^	18,728 ^a;B^	145,921 ^a;A^	53,014 ^a;B^	21,427 ^a;B^	27,076 ^g;A^
1 T18	10,458 ^d,e;A^	322,350 ^f;A^	69,180 ^c,d;A^	42,371 ^b;B^	97,513 ^a;A^	58,562 ^b;A^	10,756 ^g;B^
2 T0	1729 ^c,d;B^	54,553 ^c;B^	4161 ^d;B^	45,175 ^d;A^	19,997 ^b;B^	ND	85,764 ^e;A^
2 T18	8093 ^e;A^	299,972 ^f,g;A^	66,630 ^c,d;A^	24,523 ^d,e;B^	61,717 ^e;A^	27,634 ^e;A^	33,717 ^e,f;B^
3 T0	9960 ^b;A^	22,471 ^f;B^	2786 ^e,f;B^	18,213 ^e;B^	10,665 ^c,d;B^	ND	99,763 ^d;A^
3 T18	8527 ^e;A^	283,889 ^g;A^	88,026 ^b;A^	22,775 ^e;A^	65,826 ^d,e;A^	27,386 ^e;A^	38,825 ^e;B^
4 T0	10,595 ^b;A^	44,554 ^d;B^	3931 ^d,e;B^	19,381 ^e;B^	8506 ^d,e;B^	ND	241,727 ^b;A^
4 T18	8405 ^e;B^	542,552 ^b;A^	71,086 ^c;A^	33,941 ^c;A^	68,003 ^c,d,e;A^	85,247 ^a;A^	128,554 ^b;B^
5 T0	2765 ^c,d;B^	38,251 ^d,e;B^	14,477 ^b;B^	16,151 ^e,f;B^	9810 ^d;B^	ND	270,385 ^a;A^
5 T18	10,296 ^d,e;A^	508,137 ^c;A^	102,539 ^a;A^	27,664 ^d;A^	76,551 ^b,c;A^	88,260 ^a;A^	157,101 ^a;B^
6 T0	6677 ^b,c;B^	81,114 ^b;B^	8363 ^c;B^	79,756 ^c;A^	14,170 ^c;B^	8111 ^b;B^	77,792 ^e;A^
6 T18	23,007 ^c;A^	431,199 ^d;A^	62,136 ^d;A^	53,925 ^a;B^	84,588 ^b;A^	35,289 ^c,d;A^	60,481 ^c;B^
7 T0	7076 ^b;B^	30,949 ^e,f;B^	1757 ^f;B^	10,271 ^f;B^	7336 ^d,e;B^	ND	48,310 ^f;A^
7 T18	12,495 ^d;A^	395,491 ^e;A^	53,896 ^e;A^	36,615 ^c;A^	73,652 ^c,d;A^	39,376 ^c;A^	27,033 ^f;B^
8 T0	7992 ^b;B^	73,734 ^b;B^	8189 ^c;B^	95,222 ^b;A^	11,426 ^c,d;B^	ND	116,165^c;A^
8 T18	27,106 ^b;A^	428,136 ^d;A^	92,359 ^b;A^	52,369 ^a;B^	77,983 ^b,c;A^	32,105 ^d;A^	48,212 ^d;B^
9 T0	175,856 ^a;A^	141,284 ^a;B^	2156 ^f;B^	2604 ^g;B^	4944 ^e;B^	1777 ^c;B^	675 ^h;A^
9 T18	39,300 ^a;B^	653,989 ^a;A^	22,916 ^f;A^	25,457 ^d,e;A^	41,981 ^f;A^	38,068 ^c;A^	ND

## Data Availability

Data is contained within the article.

## Supplementary Materials

The following supporting information can be downloaded at: www.mdpi.com/article/10.3390/antiox11030490/s1, Table S1: Results of the determination of fatty acids expressed as g/100 g of oil. Data are reported as mean ± standard deviation of 4 injections of 2 independent replicates. Different letters in rows indicate statistically significant differences between time 0 and time 18 (one-way ANOVA, *p* < 0.05, Tukey’s HSD); Table S2: Volatiles detected along the heat treatment for the nine types of oil samples. Results are expressed in area counts ×10^3^/g for each compound; the ion used for the quantification of several compounds is indicated, as well as the Kovats index for each volatile; Table S3: Results of the PVs, TBARs, total phenolic content, classes of volatiles, tocopherols and CBDA/CBD ratio before submitting samples to the accelerated test. Different letter in columns indicates statistically significant differences among samples (one-way ANOVA, *p* < 0.05, Tukey HSD). Figure S1. Absorbance measured at 390 and 532 nm for samples at time 0 (a) and at time 18 (b).
