# Supplementary material for: Evaluation of Hemp Seed Oils Stability under Accelerated Storage Test
Source: Antioxidants (Basel). 2022 Feb 28;11(3):490. doi: 10.3390/antiox11030490 (PMC8944499; doi:10.3390/antiox11030490)
Supplement: Supplementary file 1 [file antioxidants-11-00490-s001.zip › antioxidants-1613260-supplementary.pdf]

Supplementary materials

Table S1. Results of the determination of fatty acids expressed as g/100 g of oil. Data are reported as mean±standard deviation of 4 injections of 2 independent replicates. Different letter in row indicates statistically significant differences between time 0 and time 18 (one-way ANOVA, $p<0.05$, Tukey's HSD).

	**Sample 1**		**Sample 2**		**Sample 3**		**Sample 4**		**Sample 5**		**Sample 6**		**Sample 7**		**Sample 8**		**Sample 9**	
**Fatty acid**	**T0**	**T18**	**T0**	**T18**	**T0**	**T18**	**T0**	**T18**	**T0**	**T18**	**T0**	**T18**	**T0**	**T18**	**T0**	**T18**	**T0**	**T18**
	**g/100 g of oil**																	
Myristic acid	0.03 ±0.00	0.03 ±0.01	0.03 ±0.00	0.03 ±0.00	0.03 ±0.00	0.02 ±0.00	0.03 ±0.00	0.03 ±0.01	0.04 ±0.00	0.04 ±0.00	0.03 ±0.00	0.03 ±0.00	0.03 ±0.00	0.03 ±0.00	0.03 ±0.00	0.03 ±0.00	0.03 ±0.00	0.03 ±0.00
Palmitic acid	5.78^{b} ±0.34	6.62^{a} ±0.08	6.16 ±0.13	6.45 ±0.07	5.89 ±0.07	6.24 ±0.24	6.24 ±0.04	5.96 ±0.27	6.30 ±0.08	6.42 ±0.02	5.43^{b} ±0.12	6.21^{a} ±0.07	6.46^{b} ±0.18	7.05^{a} ±0.14	5.45^{b} ±0.11	6.17^{a} ±0.17	5.47^{b} ±0.03	6.40^{a} ±0.08
trans-palmitoleic acid	0.03 ±0.00	0.03 ±0.00	0.03 ±0.00	0.04 ±0.00	0.03 ±0.00	0.03 ±0.00	0.03 ±0.00	0.03 ±0.00	0.03 ±0.00	0.03 ±0.00	0.03 ±0.00	0.03 ±0.00	0.03 ±0.00	0.03 ±0.00	0.03 ±0.00	0.03 ±0.00	0.03 ±0.00	0.03 ±0.00
Palmitoleic acid	0.10 ±0.01	0.11 ±0.00	0.11 ±0.00	0.11 ±0.00	0.11 ±0.00	0.11 ±0.01	0.11 ±0.00	0.10 ±0.00	0.11 ±0.00	0.11 ±0.00	0.10 ±0.00	0.11 ±0.00	0.10 ±0.00	0.11 ±0.00	0.11 ±0.00	0.11 ±0.00	0.11 ±0.00	0.12 ±0.01
Stearic acid	2.25 ±0.14	2.61 ±0.00	2.50 ±0.06	2.65 ±0.04	2.56 ±0.03	2.76 ±0.10	2.58 ±0.02	2.45 ±0.14	2.54 ±0.03	2.62 ±0.01	1.92 ±0.04	2.22 ±0.03	2.55 ±0.07	2.81 ±0.06	1.99 ±0.04	2.29 ±0.08	1.98 ±0.02	2.33 ±0.02
Oleic acid	11.46^{b} ±0.67	12.32^{a} ±0.10	10.97^{b} ±0.25	11.54^{a} ±0.07	10.84^{b} ±0.14	11.50^{a} ±0.53	13.10^{a} ±0.03	12.29^{b} ±0.57	12.88 ±0.15	13.07 ±0.12	7.05^{b} ±0.24	8.09^{a} ±0.05	11.13^{b} ±0.25	12.07^{a} ±0.20	7.67^{b} ±0.17	8.57^{a} ±0.15	8.44^{b} ±0.03	9.80^{a} ±0.07
Vaccenic acid	0.77 ±0.05	0.88 ±0.01	0.70 ±0.02	0.73 ±0.01	0.67 ±0.01	0.71 ±0.03	0.82 ±0.00	0.78 ±0.04	0.84 ±0.01	0.86 ±0.01	0.75 ±0.02	0.85 ±0.01	0.65 ±0.01	0.69 ±0.01	0.75 ±0.02	0.83 ±0.01	0.77 ±0.00	0.88 ±0.01
trans-linoleic acid	0.06^{a} ±0.00	0.01^{b} ±0.00	0.09^{a} ±0.00	0.05^{b} ±0.00	0.07 ±0.00	0.07 ±0.00	0.03^{a} ±0.00	0.01^{b} ±0.00	0.04 ±0.00	0.04 ±0.01	0.06^{a} ±0.01	0.02^{b} ±0.00	0.05^{a} ±0.00	0.02^{b} ±0.00	0.04^{a} ±0.00	0.02^{b} ±0.00	0.03 ±0.00	n.d.
cis-trans linoleic acid	0.31 ±0.01	0.32 ±0.00	0.24 ±0.00	0.25 ±0.00	0.24 ±0.00	0.24 ±0.00	0.29 ±0.01	0.27 ±0.00	0.30 ±0.01	0.28 ±0.01	0.29 ±0.00	0.30 ±0.00	0.23 ±0.00	0.24 ±0.00	0.28 ±0.00	0.30 ±0.01	0.29 ±0.01	0.30 ±0.00
trans-cis linoleic acid	n.d.	n.d.	0.05 ±0.00	0.05 ±0.00	0.04 ±0.00	0.05 ±0.00	0.05 ±0.00	0.04 ±0.00	0.06 ±0.01	0.04 ±0.00	n.d.	n.d.	0.04 ±0.00	0.04 ±0.00	0.04 ±0.00	0.05 ±0.00	0.04 ±0.00	0.04 ±0.00
Linoleic acid	48.63^{b} ±3.27	50.95^{a} ±0.85	50.67^{b} ±1.06	52.05^{a} ±1.64	50.67^{b} ±0.34	52.01^{a} ±2.54	48.10^{a} ±0.42	46.25^{b} ±0.32	49.87^{a} ±0.59	47.57^{b} ±1.87	49.95^{b} ±2.37	52.32^{a} ±0.07	51.25^{b} ±1.04	53.13^{a} ±0.64	49.40^{b} ±1.20	52.32^{a} ±0.69	46.24^{b} ±0.39	51.47^{a} ±0.04

Arachidonic acid	0.72 ±0.05	0.84 ±0.01	0.57 ±0.01	0.61 ±0.01	0.58 ±0.01	0.62 ±0.03	0.72 ±0.01	0.69 ±0.04	0.71 ±0.01	0.74 ±0.01	0.75 ±0.02	0.87 ±0.01	0.59 ±0.02	0.64 ±0.02	0.76 ±0.02	0.87 ±0.03	0.74 ±0.01	0.86 ±0.01
γ-linolenic acid	2.38 ±0.21	2.60 ±0.14	0.39 ±0.01	0.37 ±0.01	0.37 ±0.02	0.38 ±0.03	1.59 ±0.06	1.37 ±0.04	1.58 ±0.02	1.35 ±0.16	3.39 ±0.34	3.83 ±0.03	0.37 ±0.01	0.37 ±0.00	3.68 ±0.12	3.53 ±0.03	3.59 ±0.02	3.85 ±0.08
Eicosenoic acid (n-9)	0.24 ±0.02	0.29 ±0.00	0.22 ±0.01	0.24 ±0.00	0.25 ±0.00	0.27 ±0.02	0.27 ±0.00	0.26 ±0.02	0.28 ±0.00	0.28 ±0.00	0.28 ±0.01	0.33 ±0.00	0.25 ±0.01	0.27 ±0.01	0.28 ±0.01	0.32 ±0.01	0.27 ±0.00	0.32 ±0.00
α-linolenic acid	11.38 ±0.89	11.51 ±0.27	14.96 ±0.25	14.47 ±0.12	15.27 ±0.17	15.28 ±0.89	10.91 ±0.30	9.75 ±0.71	11.94 ±0.14	10.61 ±0.86	15.17 ±1.23	16.73 ±0.10	15.05 ±0.31	15.02 ±0.01	17.03 ±0.48	16.82 ±0.09	15.22[b] ±0.18	16.36[a] ±0.12
Stearidonic acid	0.49 ±0.05	0.52 ±0.03	0.12 ±0.00	0.14 ±0.02	0.13 ±0.01	0.18 ±0.02	0.32 ±0.02	0.26 ±0.02	0.34 ±0.01	0.26 ±0.04	1.04 ±0.13	1.14 ±0.02	0.16 ±0.03	0.14 ±0.01	1.21 ±0.06	1.04 ±0.03	1.07 ±0.01	1.12 ±0.04
Eicosadienoic acid (n-6)	0.12 ±0.01	0.10 ±0.02	0.09 ±0.01	0.06 ±0.02	0.10 ±0.02	0.07 ±0.01	0.11 ±0.01	0.08 ±0.01	0.08 ±0.00	0.08 ±0.01	0.16 ±0.02	0.10 ±0.02	0.12 ±0.02	0.06 ±0.01	0.20 ±0.00	0.19 ±0.02	0.12 ±0.00	0.13 ±0.00
Behenic	0.18[b] ±0.02	0.24[a] ±0.01	0.10 ±0.01	0.13 ±0.01	0.19[a] ±0.03	0.12[b] ±0.02	0.23 ±0.01	0.23 ±0.02	0.23 ±0.01	0.26 ±0.00	0.21[b] ±0.00	0.28[a] ±0.00	0.11[b] ±0.01	0.14[a] ±0.01	0.22[b] ±0.00	0.26[a] ±0.00	0.21[b] ±0.01	0.28[a] ±0.01
Brassidic	0.04 ±0.00	0.05 ±0.00	0.04 ±0.00	0.04 ±0.00	0.04 ±0.00	0.04 ±0.00	0.05 ±0.00	0.04 ±0.00	0.04 ±0.00	0.05 ±0.00	0.05 ±0.00	0.05 ±0.00	0.04 ±0.00	0.04 ±0.00	0.05 ±0.00	0.05 ±0.00	0.05 ±0.00	0.05 ±0.00
Nervonic	0.04[b] ±0.00	0.09[a] ±0.01	0.03[b] ±0.00	0.08[b] ±0.00	0.03[b] ±0.00	0.07[a] ±0.00	0.04[b] ±0.00	0.08[a] ±0.01	0.03[b] ±0.00	0.10[a] ±0.00	0.04[b] ±0.00	0.07[a] ±0.00	0.03[b] ±0.00	0.07[a] ±0.00	0.07 ±0.00	0.07 ±0.00	0.04[b] ±0.00	0.07[a] ±0.00
Lignoceric	0.14[b] ±0.01	0.21[a] ±0.01	0.10 ±0.00	0.10 ±0.00	0.09[b] ±0.01	0.14[a] ±0.01	0.16[b] ±0.01	0.20[a] ±0.01	0.15[b] ±0.00	0.23[a] ±0.03	0.16[b] ±0.01	0.23[a] ±0.00	0.09 ±0.00	0.09 ±0.00	0.13[b] ±0.01	0.20[a] ±0.00	0.14[a] ±0.01	0.10[b] ±0.00
SFA	10.52	11.45	10.59	10.88	10.45	10.71	11.41	11.51	11.08	11.83	9.96	10.21	10.88	11.43	9.43	10.20	9.95	10.44
MUFA	14.82	15.15	13.62	14.05	13.46	13.87	16.64	16.59	15.96	16.93	9.83	10.05	13.58	14.16	9.87	10.52	11.30	11.81
PUFA	73.40	72.14	75.03	74.29	75.36	74.50	70.85	70.84	71.87	70.17	78.27	77.80	74.82	73.70	78.70	77.48	76.81	75.88
n-3	13.36	12.74	17.08	16.17	17.42	16.90	12.84	12.14	13.64	12.59	18.07	17.92	16.96	16.24	19.15	17.97	18.04	17.38
n-6	59.90	59.29	57.85	58.06	57.82	57.52	57.87	58.60	58.14	57.47	60.01	59.76	57.73	57.40	59.32	59.29	58.62	58.36
*n*6/*n*3	4.48	4.65	3.39	3.59	3.32	3.40	4.51	4.84	4.26	4.57	3.32	3.33	3.40	3.53	3.10	3.30	3.25	3.36
pufa/sfa	6.99	6.30	7.09	6.84	7.21	6.95	6.21	6.16	6.49	5.94	7.87	7.62	6.88	6.45	8.35	7.60	7.72	7.27
pufa+mufa/sfa	8.40	7.62	8.37	8.13	8.50	8.25	7.67	7.61	7.93	7.37	8.86	8.60	8.13	7.69	9.39	8.63	8.85	8.40
trans	0.52	0.45	0.51	0.46	0.48	0.59	0.55	0.50	0.54	0.50	0.52	0.49	0.44	0.40	0.50	0.48	0.52	0.46

nd not detected, SFA saturated fatty acids, MUFA monounsaturated fatty acids, PUFA polyunsaturated fatty acids

Table S2. Volatile detected along the heat treatment for the nine oil samples. Results are expressed in area counts ×10^3/g for each compound; the ion used for the quantification of several compounds is indicated, as well as the Kovats index for each volatile.

Volatile compound	Ion[1]	KI[2]	Sample 1							Sample 2						
			Day 0	Day 3	Day 6	Day 9	Day 12	Day 15	Day 18	Day 0	Day 3	Day 6	Day 9	Day 12	Day 15	Day 18
**Alkenes**																
1-octene		792	1277	1293	1145	1354	1412	1589	1642	251	775	1042	1398	1318	1371	251
2-(*E*)-octene		808	4616	6134	3141	4627	5746	5935	5197	722	2308	3109	3578	3714	4454	522
2-(Z)-octene		815	2004	1892	2096	2537	3047	3659	3619	1007	826	1590	2071	2766	2698	164
**Aldehydes**																
Hexanal		804	62642	69294	63908	62382	65111	70458	68678	46097	42367	52322	48300	56688	40303	47374
(*E*)-2-hex-enal		850	1195	1619	2902	6815	6589	9063	9338	876	4832	7891	8087	8470	6904	8421
Heptanal		901	4246	4734	4823	4099	4402	4364	4506	2449	2015	2673	3312	3644	2727	3202
(*E*,*E*)-2,4-hexadienal	81	909	-	544	1485	4906	4488	6143	5584	-	1880	4177	4951	4416	4773	4842
(*E*)-2-hepte-nal		954	4503	17346	41234	67046	61070	63238	61349	990	52483	73398	69376	73565	65314	61248
(*E*,*E*)-2,4-heptadienal	81	995	1259	5544	20169	45755	51556	42852	39634	1818	33207	52792	57483	54103	67017	57168
(*E*,*Z*)-2,4-heptadienal		1009	-	5529	10155	65459	65110	71301	81457	280	23462	59649	90353	118685	106074	113161

2-octenal	1056	3130	4309	8989	28801	29959	30908	32614	1277	6129	15727	24999	21993	16634	23357
Nonanal	1103	3023	2538	2483	5658	5499	6451	7322	669	1368	3352	3712	5568	2902	3607
Nonenal	1158	571	553	757	6511	8159	18210	23701	94	461	2090	3724	3476	3280	8396
2,4-nonadienal	1192	-	-	-	1133	927	1313	1714	-	-	851	1438	1204	1116	1976
(*E*,*E*)-2,4-decadienal	1291	-	-	470	5483	6373	7440	8048	-	502	2985	4414	4700	4244	6836
2,4-decadienal	1317	-	-	280	10472	11805	15846	18040	-	1073	6390	10912	11425	9224	17547
**Ketones**															
2-heptanone	890	10028	10856	9246	6034	6032	6230	5972	3326	3070	3817	3627	3759	2865	3379
1-octen-3-one	977	1223	1947	2509	3993	3653	3511	2996	-	915	1747	1812	2151	2045	1624
3-octen-2-one	1038	4481	5164	7430	12091	12407	13276	13174	-	4065	7840	9136	9058	9011	10588
(*E*,*E*)-3,5-octadien-2-one	1070	1858	6740	15865	24816	28097	21593	20348	663	14107	21482	21855	29210	29186	23179
3,5-octadien-2-one	1091	1138	1747	6373	21508	22368	23413	26688	171	6977	16722	21333	20227	22089	27858
**Alcohols**															
3-methyl-1-butanol	727	154	120	-	-	-	-	-	413	-	-	-	-	-	-
1-pentanol	762	16661	15297	12239	6503	6337	5317	4308	6527	5768	5754	5258	5604	4665	4430
1-hexanol	873	127496	106758	73273	30852	27493	21410	17800	37524	17140	9863	8067	7156	8564	5584

1-octen-3-ol		979	1608	4010	13182	21842	18746	20333	20263	709	17065	22550	21252	21931	16945	14509
**Acids**																
Propionic acid	74	726	-	-	13199	15285	14945	13531	13164	158	10719	16437	16517	24198	17579	16839
Butanoic acid	60	801	11687	16246	15639	10532	9512	8953	8666	13247	14244	11665	10075	12459	10914	8776
Pentanoic acid	60	904	6145	6327	7149	7497	7545	7370	7345	1029	2050	4143	3957	4648	3121	4181
Hexanoic acid	60	992	32923	45230	51900	59979	59261	58943	58810	4441	10707	18202	26236	20660	19617	29525
Heptanoic acid	60	1082	2259	7385	9186	10161	9096	9633	9526	213	3644	4408	4205	4986	4341	4154
**Furans derivatives**																
2-pentyl-furan	81	991	21427	12458	16002	47213	38143	56228	58561	-	9813	26200	27308	33666	16635	27634
**Terpenes**																
α-pinene		929	8604	7685	5571	2817	2650	2344	2283	36608	16750	9966	8512	7865	9372	7005
β-pinene		971	3074	2695	2596	1238	1458	1235	1251	19832	10076	6400	5776	5175	6395	4899
δ-3-carene	93	1006	949	908	778	441	415	387	358	1932	1326	962	742	669	858	632
para-cymene	93	1021	2566	2616	2456	1654	1497	1527	1523	1427	1597	1724	1913	2134	1942	5439
Limonene		1025	2971	2445	2102	1715	1633	1537	1566	17951	13712	12257	10593	10345	11033	10060
(*E*)-β -ocimene		1047	2232	1757	2200	1902	1768	1788	1797	2794	2598	2515	2421	2168	2540	2127
Terpinolene		1085	5239	3585	2495	1672	965	1467	1296	4182	4473	3855	2696	3025	3016	3093

(*E*)-caryophyllene		1420	1264	1175	1140	619	482	456	475	967	1005	858	515	695	548	375
Bergamotene (isomer)		1439	172	160	173	196	162	191	204	70	241	63	312	90	95	95

Volatile compound	Ion[1]	KI[2]	Sample 3							Sample 4						
			Day 0	Day 3	Day 6	Day 9	Day 12	Day 15	Day 18	Day 0	Day 3	Day 6	Day 9	Day 12	Day 15	Day 18
**Alkenes**																
1-octene		792	1813	1184	1296	1108	1243	1453	1585	2701	577	845	1111	1258	1260	1930
2-(E)-octene		808	4280	3929	3079	3331	4092	4385	3853	4684	2679	3437	4267	4742	3851	2197
2-(Z)-octene		815	3867	2183	2427	2181	2323	3011	3088	3209	1583	2277	2631	3080	2843	4276
**Aldehydes**																
Hexanal		804	18512	43587	39625	36235	36835	37284	42003	33222	50275	60870	70953	72552	77201	113722
(E)-2-hexenal		850	915	4321	6535	4721	5083	6138	7460	1383	5640	6589	8147	9014	9747	14231
Heptanal		901	1256	2220	1970	1764	1776	1747	2951	1249	2372	3043	3760	3596	4233	5728
(E,E)-2,4-hexadienal	81	909	62	1603	2634	2365	2454	3498	4009	236	5174	6415	6260	6185	5485	9277
(E)-2-heptenal		954	907	61914	70486	58127	55930	54667	57562	3971	81176	84493	75316	77349	71335	101564

(E,E)-2,4-heptadi-enal	81	995	1885	40069	60777	66743	62208	69072	63409	1190	51001	52411	44562	42380	33372	47128
(E,Z)-2,4-heptadi-enal		1009	85	31430	71900	64371	57550	4817	108082	3704	49285	64707	76372	75684	82854	127569
2-octenal		1056	-	7252	12287	12174	10414	14083	3208	-	20072	28284	29722	28473	28097	41185
Nonanal		1103	434	1277	2108	1734	1654	2341	3281	787	3218	5343	6624	6317	7206	11341
Nonenal		1158	149	565	1602	1378	1357	3736	7821	-	1685	5980	18355	23332	36120	62660
2,4-non-adienal		1192	-	-	-	316	404	906	1577	-	1245	2253	3023	2906	3325	4539
(E,E)-2,4-decadi-enal		1291	-	659	2834	2792	2670	416	6349	-	3436	5651	7636	7270	8080	13724
2,4-deca-dienal		1317	-	1242	6417	5709	5061	894	15717	-	5690	11602	18035	17138	21547	37008
**Ketones**																
2-hep-tanone		890	1963	3001	2689	2647	2679	2824	3290	1904	2593	2680	3376	2989	2352	5247
1-octen-3-one		977	-	854	1279	926	990	1047	1205	434	2565	3720	3185	2898	2216	2972
3-octen-2-one		1038	-	4949	7876	7697	7381	10250	12408	-	5994	7798	9360	4954	5028	7532
(E,E)-3,5-octadien-2-one		1070	823	20630	27603	36844	35491	34646	30849	1593	20383	20905	18098	16381	13645	20819

3,5-octa-dien-2-one		1091	-	9904	19406	23132	22125	33003	40272	-	13432	19390	19731	19008	21139	34514
Alcohols																
3-me-thyl-1-butanol		727	197	-	-	-	-	-	-	947	365	254	-	-	-	-
1-penta-nol		762	2162	6368	5511	4733	4680	3707	3839	3733	5191	4558	3557	3070	2647	3535
1-hexa-nol		873	15495	6748	4687	4665	5089	3491	2753	12688	3793	2714	2039	1875	1350	1530
1-octen-3-ol		979	357	19802	23069	18887	18386	15552	16182	2012	22477	24110	22675	22694	21443	28875
**Acids**																
Propi-onic acid	74	726	-	15669	20472	20102	21722	22780	21547	-	11397	12491	12037	11596	10131	14897
Butanoic acid	60	801	9493	10600	8820	9056	9849	7804	6975	4203	3293	3156	12486	3085	2881	4356
Penta-noic acid	60	904	305	1229	1755	1740	1708	2081	3781	857	2372	3277	3630	3064	12428	5201
Hexa-noic acid	60	992	866	7759	13187	13009	12098	17814	30795	3445	17302	24210	26922	23490	24189	38840
Hep-tanoic acid	60	1082	-	2173	2460	2740	2606	2489	2726	-	2263	2390,115	10269	2235	2499	4706
**Furans deriva-tives**																

2-pentyl-furan	81	991	-	9828	18992	13407	11983	16727	27386	-	20347	29070	43481	44434	60081	85247
**Terpenes**																
α-pinene		929	39742	16676	10855	11313	12656	9158	6687	70091	33273	28899	25953	24912	22770	24780
β-pinene		971	21577	10496	7881	7749	8763	6424	4889	29314	14308	7881	7749	10458	9731	12598
δ-3-carene	93	1006	1613	1078	829	870	899	686	4804	2333	829	870	1624	1502	2005	4804
para-cymene	93	1021	1531	1826	1869	1805	1646	2004	5211	7161	6552	6321	10209	5503	4862	12953
Limonene		1025	19949	15338	13172	13512	14209	11845	12086	33134	21513	18413	16817	15904	14599	20296
(E)-β -ocimene		1047	5140	4478	4313	4664	4800	3809	3413	20345	12977	11450	10313	9768	8633	13830
Terpinolene		1085	6917	6248	5511	6032	6350	4667	4280	32112	20087	15183	13610	11707	12353	18091
(E)-caryophyllene		1420	3035	3124	2510	2490	2507	1760	1424	40220	25171	17609	13715	11218	11043	17289
Bergamotene (isomer)		1439	255	617	591	332	385	303	302	4543	4707	4559	4556	4060	4224	6710

Volatile compound	Ion[1]	KI[2]	Sample 5							Sample 6						
			Day 0	Day 3	Day 6	Day 9	Day 12	Day 15	Day 18	Day 0	Day 3	Day 6	Day 9	Day 12	Day 15	Day 18
**Alkenes**																
1-octene		792	1828	651	752	941	1120	977	74	1803	6551	2475	3058	3551	3760	3874

2-(E)-octene		808	936	2143	2475	2587	3452	3029	5311	4874	2645	5946	8575	9119	10416	10730
2-(Z)-octene		815	-	-	1191	1320	2117	1790	3329	-	2083	5137	7105	8182	9056	8403
**Aldehydes**																
Hexanal		804	30184	50535	65699	76635	86329	86925	135559	65268	43505	58679	66388	61232	62012	60217
(E)-2-hexenal		850	731	3625	5409	6849	8513	9274	13882	1437	3040	7256	8333	9443	10422	10836
Heptanal		901	1415	2251	3122	3495	3885	3997	6164	2394	1985	3427	4630	4459	4168	4313
(E,E)-2,4-hexadienal	81	909	153	4695	7762	6902	6008	5045	7871	132	2043	6728	7320	8290	8005	7255
(E)-2-heptenal		954	-	50166	76728	62160	67507	52341	86424	5379	38130	77400	82767	76619	83701	82125
(E,E)-2,4-heptadienal	81	995	1117	30293	45472	40711	35613	32034	40163	1582	26727	82702	90501	89239	88743	81878
(E,Z)-2,4-heptadienal		1009	1361	24196	49169	56690	60072	60659	98646	1582	1344	20696	91259	131287	146401	152645
2-octenal		1056	3969	9451	20447	22110	21708	19919	34539	3070	7865	25155	39953	32497	34397	36152
Nonanal		1103	681	2793	5618	6850	7745	7418	12612	1510	1531	3355	5329	5452	5691	6174
Nonenal		1158	-	-	3981	17202	32996	42259	76043	342	546	2597	6015	8091	9978	13503
2,4-nonadienal		1192	-	653	1956	350	2463	2335	4134	-	-	-	-	-	-	-

(E,E)-2,4-decadienal	1291	-	-	3526	252	5209	5303	9018	-	674	6494	10145	11443	12164	13664
2,4-decadienal	1317	-	691	6411	412	11617	12747	23246	-	580	9452	18965	22307	24000	28665
**Ketones**															
2-heptanone	890	2774	3272	3642	3795	3920	3685	5305	4133	3713	4775	5539	5174	5264	5268
1-octen-3-one	977		2336	4155	3109	3632	2111	3126	670	1744	5130	6345	5831	5438	670
3-octen-2-one	1038	8830	14920	19857	19729	19248	17501	25385	3560	5073	11159	13128	13666	13395	14816
(E,E)-3,5-octadien-2-one	1070	2874	32801	38290	34437	28264	26673	35660	-	13663	33987	30987	32474	29680	28451
3,5-octadien-2-one	1091	-	17070	26442	30243	29318	30739	51107	-	5144	5792	7397	6391	7756	8163
**Alcohols**															
3-methyl-1-butanol	727	651	-	-	-	-	-	-	309	-	-	-	-	-	-
1-pentanol	762	3644	4367	4178	3624	3044	2455	3165	7892	6329	8002	7680	7468	6475	6057
1-hexanol	873	9284	3635	2039	1572	1254	949	1112	69264	39831	32579	24861	24459	22543	19254
1-octen-3-ol	979	2572	12478	22154	19264	20222	16113	23387	2290	13513	29462	30129	28770	29305	28613

**Acids**																
Propionic acid	74	726	-	12626	14204	13957	9988	12532	17467	-	5310	16659	18356	19523	19563	18378
Butanoic acid	60	801	3949	3444	3404	3295	3313	3031	4589	3713	7184	9811	9575	9506	9271	8409
Pentanoic acid	60	904	1079	2656	3682	3897	4040	3638	5929	1429	2372	4582	7113	6214	6351	6341
Hexanoic acid	60	992	4592	17432	26420	28720	28559	27045	42971	8901	15882	33774	46393	43028	43955	46407
Heptanoic acid	60	1082	190	2713	3437	3493	3369	3437	5595	127	2294	4573	5005	4699	4838	5053
**Furans derivatives**																
2-pentylfuran	81	991	-	-	28035	40424	49725	54352	88260	8112	10601	24205	33614	33640	33493	35289
**Terpenes**																
α-pinene		929	81883	50323	38111	34399	32598	31246	32083	25534	16780	16202	12999	12753	12760	11578
β-pinene		971	34338	21088	33977	14210	13524	12578	17188	11673	8979	8566	7352	6981	6720	5898
δ-3-carene	93	1006	5332	3454	2383	2183	2052	1886	2508	4293	3386	3441	2739	2756	2714	2490
para-cymene	93	1021	8861	8344	12155	11202	11150	10108	15196	2403	2471	2737	7913	8437	8023	7591
Limonene		1025	37132	28761	23595	21941	18755	17969	24241	12974	11255	13393	13903	13036	12983	15517

(E)-β -ocimene		1047	-	15950	13635	12468	11328	10283	14804	8112	6624	8362	7126	7058	6987	6633
Terpino-lene		1085	27185	21154	13141	12752	11246	11995	16788	11500	10156	12281	10230	11108	10329	9709
(E)-cary-o-phyllene		1420	53095	39526	25080	20313	16643	15627	24710	1304	1212	1598	1387	1261	1103	1065
Berga-motene (isomer)		1439	6050	6402	6602	6500	6016	5804	9584	-	-	-	-	-	-	-

Volatile compound	Ion[1]	KI[2]	Sample 7							Sample 8						
			Day 0	Day 3	Day 6	Day 9	Day 12	Day 15	Day 18	Day 0	Day 3	Day 6	Day 9	Day 12	Day 15	Day 18
**Alkenes**																
1-octene		792	378	761	1521	1997	2287	2372	2557	1946	1499	2640	3293	3133	3985	4388
2-(E)-oc-tene		808	3839	2821	4207	4022	4796	5086	5613	3810	2447	5999	7828	8410	11830	12774
2-(Z)-oc-tene		815	2859	1322	3100	3325	4097	4243	4325	2235	2024	5001	6808	7640	9393	9944
**Alde-hydes**																
Hexanal		804	24168	36898	46876	48812	51229	50944	52120	60005	51230	54850	54741	57499	58360	59456
(E)-2-hexenal		850	432	3872	9704	10950	11847	11462	11856	1837	3997	6698	7637	8128	10119	10957
Heptanal		901	1255	1776	2402	3537	3783	3576	3927	2227	2280	3112	2974	3516	3870	4177

(E,E)-2,4-hexadi-enal	81	909	-	1148	4068	5170	5355	4683	5428	-	3562	8007	8490	8305	9347	8200
(E)-2-heptenal		954	2140	23761	63186	75377	78766	75165	77298	5134	27579	58354	69304	78179	79883	79724
(E,E)-2,4-heptadi-enal	81	995	9979	40753	87515	85008	80969	80211	72908	946	36200	81852	90816	89396	88156	83870
(E,Z)-2,4-heptadi-enal		1009	1875	33626	112457	151247	159055	165147	165689	-	23797	90460	119781	135477	161530	169121
2-octenal		1056	1079	7334	16878	23822	25219	23744	23372	2614	8049	25704	28423	35442	34154	35708
Nonanal		1103	-	1275	2957	4180	4345	4266	3974	971	1517	3405	4560	5160	6157	6410
Nonenal		1158	-	244	2171	5635	7905	8471	10446	-	-	2107	4150	6520	10393	13011
2,4-non-adienal		1192	-	-	-	-	2040	2284	2178	-	-	1297	2095	2186	2860	2892
(E,E)-2,4-decadi-enal		1291	-	646	5147	8937	9485	9968	9939	-	-	5788	9226	10514	12393	12213
2,4-deca-dienal		1317	-	1132	11904	24118	27321	27806	29263	-	-	8760	11197	19088	25023	26269
**Ketones**																
2-hep-tanone		890	-	-	-	-	-	-	-	4521	4124	4245	3996	4735	4748	5080
1-octen-3-one		977	-	751	2689	2110	2259	2223	1994	630	2841	6662	7158	5882	5621	6199

3-octen-2-one		1038	-	5439	12869	15604	16346	17361	17096	3038	5422	10420	11890	12584	14655	14841
(E,E)-3,5-octadien-2-one		1070	1757	21059	37683	33590	31161	33649	29854	-	21198	33109	35257	34797	32023	29639
3,5-octa-dien-2-one		1091	-	1972	3901	4487	5340	5823	4952	-	-	5299	27034	31531	36711	36599
**Alcohols**																
3-me-thyl-1-butanol		727	355	992	1942	1314	2415	4197	2240	878	653	2740	2354	2185	2545	2561
1-penta-nol		762	2559	4737	5931	5592	5691	5192	5053	12016	8386	7701	6626	6379	6035	5372
1-hexa-nol		873	7356	3546	3720	3035	3002	2927	2868	80186	42281	21665	18282	16293	15178	14127
1-octen-3-ol		979	-	16919	32162	30591	29827	27681	26454	2142	17502	30694	29852	28183	29956	30309
**Acids**																
Propi-onic acid	74	726	-	13735	25075	26391	25235	27130	25266	-	7596	16197	18794	20044	20632	20114
Butanoic acid	60	801	6339	9416	11484	10373	10263	10343	10155	5426	8859	8158	8037	7736	8088	7879
Penta-noic acid	60	904	190	1684	3678	5031	5342	5130	4980	1194	2180	4157	4391	5219	5339	5660
Hexa-noic acid	60	992	808	7875	19197	28634	30327	30732	30282	4806	13661	27517	32232	37820	38863	40626

Heptanoic acid	60	1082	-	1739	3623	3344	3395	3295	2968	-	2297	3082	3623	3682	3643	3705
**Furans derivatives**																
2-pentyl-furan	81	991	-	-	24547	37736	40499	37063	39376	-	-	19575	23073	27203	28120	32105
**Terpenes**																
α-pinene		929	21406	8292	8398	7109	6738	6808	6507	50728	27569	15353	13131	12761	11375	11467
β-pinene		971	10351	5330	5966	5057	5041	5095	4523	17312	11005	6722	5522	5636	4921	4789
δ-3-carene	93	1006	1509	1001	1097	919	862	889	836	11113	8034	4967	4414	4121	3867	3766
para-cymene	93	1021	938	941	1465	1608	1621	1640	1499	3698	4220	3994	3700	9919	10572	9325
Limonene		1025	8737	7763	9698	8924	8775	8993	8040	18220	16025	12504	11572	11829	10889	10328
(E)-β -ocimene		1047	-	-	-	-	-	-	-	13406	12259	9717	9981	9806	7904	7717
Terpinolene		1085	4346	3727	4865	4463	4461	4685	4766	-	-	-	-	-	-	-
(E)-caryophyllene		1420	1023	980	1352	1132	1032	1009	862	1689	1679	1248	1206	1084	919	820
Bergamotene (isomer)		1439	-	-	-	-	-	-	-	-	-	-	-	-	-	-

Volatile compound	Ion[1]	KI[2]	Sample 9						
			Day 0	Day 3	Day 6	Day 9	Day 12	Day 15	Day 18
**Alkenes**									
1-octene		792	21722	8095	4945	4387	4750	4845	4653
2-(E)-octene		808	83333	31320	14022	17435	18800	20483	19748
2-(Z)-octene		815	70800	25639	15690	13620	13949	15204	14898
**Aldehydes**									
Hexanal		804	46589	52164	65886	86575	87469	102370	111396
(E)-2-hexenal		850	3893	4147	3758	1214	7265	10031	12534
Heptanal		901	2974	2231	2421	2708	2871	2918	3722
(E,E)-2,4-hexadienal	81	909	-	1658	5366	8520	8717	10242	11128
(E)-2-heptenal		954	73582	85798	73737	65516	60403	63992	65532
(E,E)-2,4-heptadienal	81	995	41761	99891	137971	127719	130425	121482	85322
(E,Z)-2,4-heptadienal		1009	9402	48862	113429	166438	168800	193527	216637

2-octenal	1056	2304	27628	94503	133690	145154	153966	140736
Nonanal	1103	116	2726	3703	5837	5882	6341	6846
Nonenal	1158	-	1644	4005	5678	15996	22773	35679
2,4-non-adienal	1192	-	-	-	-	-	-	-
(E,E)-2,4-decadi-enal	1291	-	4612	10720	16151	17418	16500	15784
2,4-deca-dienal	1317	-	3578	8669	25742	26684	27589	33994
**Ketones**								
2-hep-tanone	890	658	2129	-	-	-	-	-
1-octen-3-one	977	1498	1331	2692	2671	2027	1725	1135
3-octen-2-one	1038	-	-	-	-	-	-	-
(E,E)-3,5-octadien-2-one	1070	-	6107	10854	13154	13577	13258	9353
3,5-octa-dien-2-one	1091	-	1860	5527	8697	8909	10079	12428
**Alcohols**								
3-me-thyl-1-butanol	727	-	-	-	-	-	-	-

1-pentanol		762	553	-	-	-	-	-	-
1-hexanol		873	1304	1468	1462	1214	1211	1294	1333
1-octen-3-ol		979	746	17601	22170	23444	20931	22510	24124
**Acids**			-						
Propionic acid	74	726		3561	7260	8935	8932	10049	8685
Butanoic acid	60	801	3497	4558	4129	4009	3745	4129	3811
Pentanoic acid	60	904	408	1333	1967	2354	2624	2711	2553
Hexanoic acid	60	992	1039	5871	13686	87220	23750	24728	24085
Heptanoic acid	60	1082	-	1968	2798	3261	3464	2939	2845
**Furans derivatives**									
2-pentylfuran	81	991	1777	7193	13858	20997	19745	25239	38068
**Terpenes**									
α-pinene		929	675	-	-	-	-	-	-
β-pinene		971	-	-	-	-	-	-	-

δ-3-carene	93	1006	-	-	-	-	-	-	-
para-cymene	93	1021	-	-	-	-	-	-	-
Limonene		1025	-	-	-	-	-	-	-
(E)-β -ocimene		1047	-	-	-	-	-	-	-
Terpinolene		1085	-	-	-	-	-	-	-
(E)-caryo-phyllene		1420	-	-	-	-	-	-	-
Bergamotene (isomer)		1439	-	-	-	-	-	-	-

Table S3. Results of the PVs, TBARs, Total Phenolic content, classes of volatiles, tocopherols and CBDA/CBD ratio before submitting samples to the accelerated test. Different letter in columns indicates statistically significant differences among samples (one-way ANOVA, $p<0.05$, Tukey HSD).

Sample	PVs (mEqO_2/kg of oil)	TBARs (mg MDA/kg of oil)	Aldheydes (area counts $\times10^3$)	Ketones (area counts $\times10^3$)	Acids (area counts $\times10^3$)	Furans (area counts $\times10^3$)	Terpenes (area counts $\times10^3$)	Total phenols (mg gallic acid/kg of oil)
1	12.62±1.07^{a}	11.28±0.69^{a}	79310^{b}	18728^{a}	53014^{a}	21427^{a}	27076^{g}	50.37±2.03^{c}
2	11.78±0.60^{a,b}	11.04±1.58^{a}	54553^{c}	4161^{d}	19997^{b}	-	85764^{e}	106.50±8.8^{b}
3	9.43±0.25^{c}	13.27±1.78^{a}	22471^{f}	2786^{e,f}	10665^{c,d}	-	99763^{d}	45.58±2.71^{c}
4	11.41±0.66^{a,b}	11.56±1.70^{a}	44554^{d}	3931^{d,e}	8506^{d,e}	-	241727^{b}	15.39±0.79^{e}
5	10.42±0.54^{b,c}	11.54±0.28^{a}	38251^{d,e}	14477^{b}	9810^{d}	-	270385^{a}	16.75±0.41^{e}
6	11.93±0.88^{a,b}	11.37±1.57^{a}	81114^{b}	8363^{c}	14170^{c}	8111^{b}	77792^{e}	30.76±6.60^{d}
7	10.87±0.41^{a,b,c}	12.25±1.15^{a}	30949^{e,f}	1757^{f}	7336^{d,e}	-	48310^{f}	25.70±1.07^{d}
8	11.95±0.81^{a,b}	12.01±0.97^{a}	73734^{b}	8189^{c}	11426^{c,d}	-	116165^{c}	12.08±1.96^{e}

9	1.83±0.06^{d}	17.15±0.83^{a}	141284^{a}	2156^{f}	4944^{e}	1777^{c}	675^{h}	186.78±4.5^{a}

Sample	C18:1 (g/100 g of oil)	C18:2 n-6 (g/100 g of oil)	C18:3 n-3 (g/100 g of oil)	C18:3 n-6 (g/100 g of oil)	γ-tocopherol (μg/g)	α-tocopherol (μg/g)	CBDA/CBD
1	11.50^{b}	48.62^{a,b,c}	11.40^{c}	2.43^{b}	641.91±29.25^{b}	32.09±0.78^{b,c}	1.10^{d}
2	10.90^{c}	50.37^{a,b}	14.89^{b}	0.39^{d}	568.68±39.34^{b}	28.28±0.85^{b,c}	-
3	10.80^{c}	50.58^{a,b}	15.31^{b}	0.37^{d}	569.99±16.46^{b}	45.98±4.15^{b,c}	6.58^{a}
4	13.08^{a}	48.20^{a,b,c}	10.99^{c}	1.61^{c}	376.28±10.72^{c}	21.02±0.28^{c}	2.27^{b,c}
5	12.92^{a}	50.04^{a,b}	11.98^{c}	1.58^{c}	650.52±14.68^{b}	41.53±3.09^{b,c}	2.62^{b,c}
6	7.12^{f}	47.63^{b,c}	15.53^{b}	3.51^{a}	906.63±65.75^{a}	65.92±4.86^{b}	-
7	11.05^{b}	50.96^{a}	14.96^{b}	0.37^{d}	622.24±12.12^{b}	59.30±1.46^{b,c}	1.72^{c,d}
8	7.72^{e}	49.76^{a,b}	17.17^{a}	3.71^{a}	893.81±7.78^{a}	49.81±0.63^{b,c}	2.17^{b,c}
9	8.45^{d}	46.35^{c}	15.27^{b}	3.59^{a}	816.31±53.49^{a}	1095.62±45.21^{a}	1.44^{d}

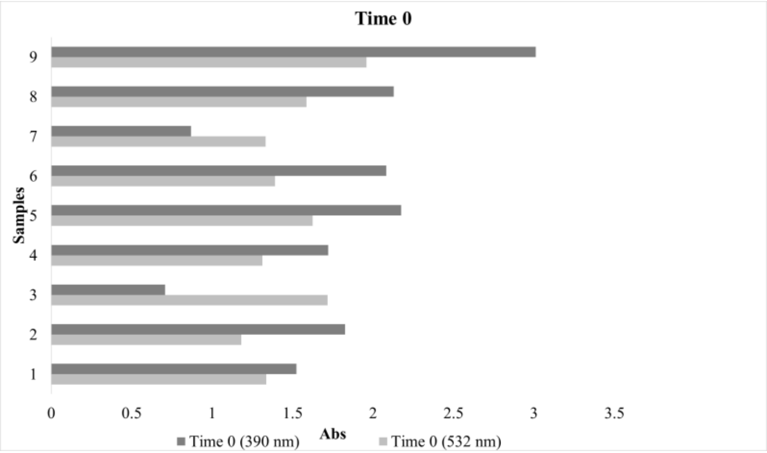

(a)

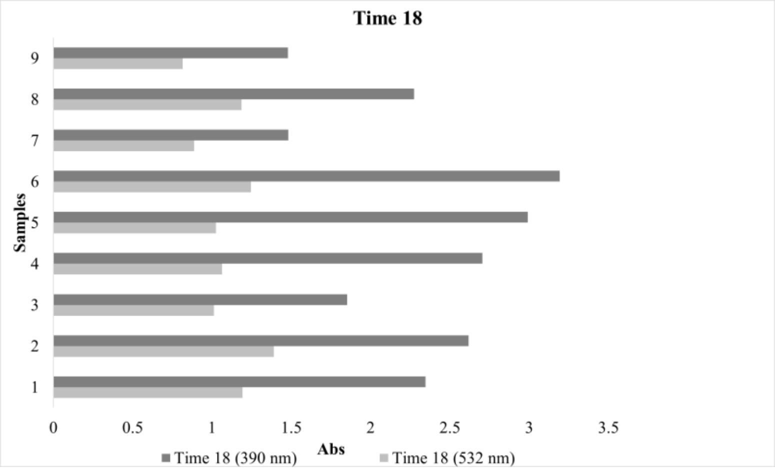

(b)

Figure S1. Absorbance measured at 390 and 532 nm for samples at time 0 (a) and at time 18 (b).
